# Mortality of Urban Aboriginal Adults in Canada, 1991–2001* *This article is part of a joint publication initiative between *Preventing Chronic Disease* and *Chronic Diseases in Canada*. *Preventing Chronic Disease* is the secondary publisher, while *Chronic Diseases in Canada* is the primary publisher.


**Published:** 2010-12-15

**Authors:** Michael Tjepkema, R. Wilkins, S. Senécal, É. Guimond, C. Penney

**Affiliations:** Health Analysis Division Statistics Canada; Health Analysis Division, Statistics Canada, Ottawa, Ontario. Department of Epidemiology and Community Medicine, University of Ottawa, Ottawa, Ontario; Strategic Research and Analysis Directorate, Indian and Northern Affairs Canada, Gatineau, Quebec. Department of Sociology, University of Western Ontario, London, Ontario; Strategic Research and Analysis Directorate, Indian and Northern Affairs Canada, Gatineau, Quebec. Department of Sociology, University of Western Ontario, London, Ontario; Strategic Research and Analysis Directorate, Indian and Northern Affairs Canada, Gatineau, Quebec

## Abstract

**Objective:**

To compare mortality patterns for urban Aboriginal adults with those of urban non-Aboriginal adults.

**Methods:**

Using the 1991–2001 Canadian census mortality follow-up study, our study tracked mortality to December 31, 2001, among a 15% sample of adults, including 16 300 Aboriginal and 2 062 700 non-Aboriginal persons residing in urban areas on June 4, 1991. The Aboriginal population was defined by ethnic origin (ancestry), Registered Indian status and/or membership in an Indian band or First Nation, since the 1991 census did not collect information on Aboriginal identity.

**Results:**

Compared to urban non-Aboriginal men and women, remaining life expectancy at age 25 years was 4.7 years and 6.5 years shorter for urban Aboriginal men and women, respectively. Mortality rate ratios for urban Aboriginal men and women were particularly elevated for alcohol-related deaths, motor vehicle accidents and infectious diseases, including HIV/AIDS. For most causes of death, urban Aboriginal adults had higher mortality rates compared to other urban residents. Socio-economic status played an important role in explaining these disparities.

**Conclusion:**

Results from this study help fill a data gap on mortality information of urban Aboriginal people of Canada.

**Keywords:**

Aboriginal people, First Nations, Métis, Inuit, North American Indians, age-standardized mortality rates, mortality rate, life expectancy

## Introduction

The number of Aboriginal people (First Nations, Métis and Inuit) living in urban Canada has increased dramatically over the last half-century; in 1950, about 7% resided in urban Canada,^1^ but by 2006 that figure had risen to 54%.^2^ However, the amount of health research on urban Aboriginal people is not proportional to their weight in the total population;^3,4^ nor does it reflect their increasing proportion within the total Aboriginal population.

Aboriginal people choose to live in urban areas for various reasons, including family reasons, employment opportunities, education, training and health (for example, to be closer to medical services);^5,6^ they face different challenges to their rural counterparts, such as finding adequate housing and locating existing services and support to assist them in the transition.^5,7^

Although it is widely known that, compa­red to other Canadians, Aboriginal people experience a disproportionate burden of death and disease,^8-12^ specific infor­mation for those residing in urban areas is less well known.^13^ Similarly, while overall life expectancy for First Nations, Métis and Inuit is considerably shorter than that of the general population,^14-18^ mortality indicators for Aboriginal people residing in urban Canada are difficult to estimate because Aboriginal identifiers are not reported on death registrations in most provinces. Mortality patterns for Registered Indians living in Manitoba and British Columbia have been analysed and provide results for sub-provincial regions including Winnipeg^19^ and Vancouver.^20^ However, these studies only show part of the picture, as they exclude First Nations not registered under the *Indian Act*, as well as Métis and Inuit, and they provide no information specific to Aboriginal people living in other urban areas of Canada.

The 1991–2001 Canadian census mortality follow-up study provides an opportunity to examine patterns of mortality for a reasonably large number of Aboriginal people living in urban areas *at the beginning of the follow-up period* in all provinces and territories, regardless of whether they were registered under the *Indian Act*.

The objectives of this study are (1) to determine to what extent urban Aboriginal adults may be at risk of premature mortality; (2) to calculate life expectancy and probability of survival to age 75 years; and (3) to identify the causes of death with the highest risk.

## Methods

### Data sources

The Canadian census mortality follow-up study consists of a 15% sample (n = 2 735 152) of the non-institutionalized population of Canada aged 25 years or older, all of whom were enumerated via the 1991 census long–form questionnaire. This cohort was tracked for mortality from June 4, 1991, to December 31, 2001. Briefly, the creation of the census mortality database required two linkages because the electronic files of census data contained no names, but names were needed to find the corresponding deaths. Using common variables such as date of birth, postal code, plus spousal date of birth (if applicable), the census file was first probabilistically linked to an encrypted name file abstracted from non-financial tax-filer data. Then this census plus encrypted name file was matched to the Canadian Mortality Database using probabilistic record linkage methods^21^—an approach similar to that used for other mortality follow-up studies at Statistics Canada.^22^ Complete details of the construction and contents of the linked file are reported elsewhere.^16^

### Eligibility

Only people who were enumerated by the 1991 census long-form questionnaire, were 25 years old by census day, and were Canadian residents were eligible to be part of the cohort. Data quality reports estimated that the 1991 census missed 3.4% of Canadian residents of all ages. The missed individuals were more likely to be young, mobile, low income, of Aboriginal ancestry^23, 24^ or homeless. Only cohort members living in urban areas (defined below) on census day were in the scope of this study. The long-form census questionnaire is usually given to one in five Canadian households, to all residents of Indian Reserves, to all residents of many remote and northern communities and to all residents of non-institutional collective dwellings. In addition, it was necessary to obtain encrypted names from tax filer data (the name file), as only tax filers could be followed for mortality. However, there were no major differences in demographic and socio-economic characteristics between eligible census respondents and those successfully linked to the name file (Appendix Tables A, B and C).

### Analytical techniques

For each member of the cohort, we calculated person-days of follow-up from the beginning of the study (June 4, 1991) to the date of death or emigration (ascertained from the name file and known for 1991 only) or the end of study (December 31, 2001). To calculate person-years at risk, we divided person-days of follow-up by 365.25.

Using the total Aboriginal† cohort population structure (person-years at risk) as the standard population, we used age- and sex-specific mortality rates by 5-year age groups (at baseline) to calculate age-standardized mortality rates (ASMRs) for subgroups of the population. We calculated corresponding 95% confidence intervals (CIs) for the ASMRs as described by Carrière and Roos,^25^ and used a similar method to calculate CIs for the ASMR ratios (RRs).

For age-specific analyses, cohort members were categorized by 10-year age groups at baseline from 25-to-34 to 65-to-74, and 75 or older. Most analyses used age baseline (June 4, 1991), while life table analyses used age at the beginning of each year of follow-up.

Based on Chiang's method,^26^ we calculated period life tables for each sex, with corresponding standard errors and 95% CIs. We calculated these after converting from age at baseline to age at the beginning of each year of follow-up, and then calculated deaths and person-years at risk separately for each year (or partial year) of follow-up. We then pooled deaths and person-years at risk by age at the beginning of each year of follow-up, before calculating the life tables.

We calculated Cox proportional mortality hazard ratios by sex, first controlling for age (in years) and then controlling for place of residence (metropolitan areas, smaller urban centres), lone parent (yes, no), education (less than high school diploma, high school diploma, post-secondary diploma, university degree), income quintile (1–5), occupation skill level (professional, managerial, skilled-technical-supervisory, semiskilled, unskilled, no occupation), work status (employed, unemployed, not in labour force) and place of birth (Canada or elsewhere). Place of birth was included in the models to reduce the "healthy immigrant effect" among non-Aboriginal cohort members. Note that detailed definitions of these variables (all ascertained only at baseline) have been previously described.^16^ We interpreted differences in excess mortality between the age-adjusted model and the fully adjusted model as estimates of the effect of the socio-economic variables (place of residence, lone parent, education, etc., as listed above) on the extent of the disparities between urban Aboriginal adults and urban non-Aboriginal adults. The proportion of excess mortality attributed to the socio-economic variables was calculated as follows: the difference between age-adjusted and fully adjusted hazard ratios for Aboriginal person (yes/no), divided by the age-adjusted hazard ratio minus 1.

The underlying cause of death of those who died in the period 1991 through 1999 was coded based on the World Health Organization's *International Classification of Diseases, Ninth Revision* (ICD-9)^27^ and those who died in 2000 or 2001 based on the *Tenth Revision* (ICD-10).^28^ For analyses by cause of death, deaths were grouped by ICD-9 chapter, categories within chapters, and by risk factors (smoking-related, alcohol-related, drug-related, or amenable to medical intervention).^29,30^ This data is presented in Appendix Table D.

†Anyone who indicated North American Indian, Métis or Inuit ancestry, Registered Indian status or membership in a North American Indian band or First Nation in the long-form census questionnaire—see Definitions.

### Definitions

The 1991 census did not collect information on self-identification with an Aboriginal group (North American Indian, Métis or Inuit). For our analysis, we defined this population on the basis of two questions, offering three distinct dimensions of Aboriginality:

1. Ancestry: Question 15 from the long-form census questionnaire asked respondents to check from a list of 15 the ethnic or cultural group(s) their ancestors belonged to, including North American Indian, Métis and Inuit/Eskimo.^31^ Respondents were instructed to specify as many as applicable.
2. Registered Indian status: Question 16 from the long-form census questionnaire asked, "Is this person a Registered Indian as defined by the *Indian Act* of Canada?" (Yes, No).
3. Indian band/First Nation membership: Question 16 also asked respondents to write down the name of the Indian band or First Nation to which they belonged.

For our study, a person was considered Aboriginal if they reported a single Aboriginal—and no other—ancestry or two or more Aboriginal ancestries (with or without any non-Aboriginal ancestry), or if they reported that they were a Registered Indian or member of an Indian band or First Nation. Based on an analysis of 1996 census data where ethnic origins were cross-classified by Aboriginal identity,^32^ over 94% of 1996 censusparticipants who met these ancestry-based definitions self-identified as Aboriginal. The number of Aboriginal people (especially Métis) would be underestimated in our study since persons reporting one Aboriginal ancestry but at least one non-Aboriginal ancestry were considered as non-Aboriginal (unless they indicated being a registered Indian or member of an Indian band or First Nation).

"Urban areas" can be defined differently depending on the research question and data availability,^33^ and our definition differs from the standard census definition.^31^ We defined "urban areas" as any census metropolitan area ("metropolitanareas," with a population of ≥ 100 000) or census agglomeration ("smaller urban centres," with a population of ≥ 10 000), excluding reserves or other Aboriginal settlements within those areas. Other urban areas were out of the scope of this study.

### Cohort members, linkage rates, deaths and person-years at risk

Appendix Table A shows that there were 2.6 million eligible census respondents in urban areas of Canada, including 25 500 Aboriginal adults. Linkage rates to the name file (comparing cohort members to long-form census respondents) for urban Aboriginal people (61% for men and 66% for women) were lower than for the urban non-Aboriginal population (80% for men and 76% for women). Despite the lower linkage rate, the demographic and socio-economic characteristics of urban Aboriginal cohort members were generally similar to those of all eligible (in-scope) urban Aboriginal adults in the weighted census population, with the following exceptions: persons who were employed, those with higher household income adequacy, and those who were married were slightly more likely to be successfully linked (a similar finding to that for non-Aboriginal cohort members), suggesting that our sample of urban Aboriginal people was not biased with respect to those characteristics (Appendix B and C).

Based on deaths in 1991, which could be identified independently in the Canadian Mortality Database and/or the name file, we estimated that ascertainment of deaths in the cohort followed for mortality (1991–2001) was about 97% overall and about 95% to 96% among Aboriginal people.

Overall, the cohort followed for mortality included 16 300 urban Aboriginal adults who accounted for 166 570 person-years at risk and 1126 deaths during the 11-year follow-up period (Appendix Table A).

## Results

According to the 1991 census, there were an estimated 259 800 Aboriginal persons aged 25 or older, representing 1.5% of the total adult population of Canada. About 45% lived in urban areas (30% in metropolitan areas, 15% in smaller urban centres). In comparison, 78% of non-Aboriginal persons lived in urban areas (62% in metropolitan areas, 16% in smaller urban centres). In all urban areas taken together, 69% of the Aboriginal population were First Nations (40% Registered Indians, 29% non-status Indians), 28% Métis and 3% Inuit.

There were 16 300 Aboriginal cohort members residing in either a metropolitan area or a smaller urban centre at the beginning of the follow-up period (June 4, 1991). Table 1 shows the demographic and socio-economic characteristics for Aboriginal and non-Aboriginal cohort members residing in urban Canada. Almost three-quarters of Aboriginal cohort members were aged 25 to 44 years compared to 54% for non-Aboriginal adults. About 44% of Aboriginal adults had less than a high school diploma (31% for non-Aboriginal adults) and 61% were in the two lowest income adequacy quintiles (36% for non-Aboriginal adults).

### Remaining life expectancy at age 25 years and probability of survival from ages 25 to 75 years

For urban Aboriginal adults of both sexes, remaining life expectancy at age 25 years (conditional on surviving to age 25 years) was substantially shorter compared to urban non-Aboriginal adults. Table 2 shows that life expectancy at age 25 years for urban Aboriginal men was 48.1 years (95% CI: 47.1-49.1), compared to 52.8 years (95% CI: 52.8-52.9) for urban non-Aboriginal men, a difference of 4.7 years. Life expectancy at age 25 years for urban Aboriginal women was longer than that for urban Aboriginal men, but the gap between the life expectancy of urban Aboriginal women (52.7 years; 95% CI: 51.7-53.7) and urban non-Aboriginal women (59.2 years; 95% CI: 59.2-59.3) was larger (6.5 years). Life expectancy for Aboriginal adults residing in metropolitan areas was similar to that for Aboriginal adults residing in smaller urban centres.

Table 2 also shows the probability of sur­vival to age 75 years, conditional on survival to age 25 years, for urban cohort members. About 52% (95% CI: 48-56) of urban Aboriginal men were expected to survive to age 75 years compared to 65% (95% CI: 64-65) of urban non-Aboriginal men, a difference of 12 percentage points. For urban Aboriginal women, 63% (95% CI: 59-66) were expected to survive to age 75 years, compared to 80% (95% CI: 79-80) for urban non-Aboriginal women, a difference of 17 percentage points.

### Age-specific and age-standardized mortality rates

Table 3 shows age-specific and age-standardized mortality rate ratios (RRs) for urban Aboriginal adults compared to urban non-Aboriginal adults. Overall, rate ratios were significantly higher for urban Aboriginal men (RR = 1.56; 95% CI: 1.43-1.70) and women (RR = 1.94; 95% CI: 1.78-2.11) compared to urban non-Aboriginal men and women. For urban Aboriginal adults of both sexes, rate ratios were highest in the younger age groups and diminished with advancing age.

### Causes of death

Table 4 shows ASMRs by major causes of death for urban Aboriginal cohort members while Table 5 shows ASMRs by major causes of death for urban non-Aboriginal cohort members. Among urban Aboriginal men, the most common causes of death were circulatory system diseases (accounting for 33% of the total ASMR), followed by all cancers (23%) and external causes (16%)—a similar ranking to that for urban non-Aboriginal men. For urban Aboriginal women, the most common causes of death were circulatory system diseases (29% of the total ASMR), followed by all cancers (26%), external causes (10%) and digestive system diseases (9%); for urban non-Aboriginal women, cancer was the most common cause of death (42%), followed by circulatory system diseases (29%), respiratory system diseases (6%) and external causes (6%).

Table 5 shows age-standardized rate ratios (RRs) by major causes of death. (The corresponding number of deaths and ASMRs are shown in Table 4 and Appendix Table D.) Rate ratios for urban Aboriginal men were substantially elevated for deaths due to circulatory system diseases (RR = 1.50; 95% CI: 1.29-1.74) such as ischemic heart disease (RR = 1.52; 95% CI: 1.26-1.83), but not for all cancers combined (RR = 1.09; 95% CI: 0.92-1.30); however, the rate ratio was elevated for deaths due to trachea, bronchus and lung cancer (RR = 1.42; 95% CI: 1.08-1.88) especially for Aboriginal men living in metropolitan areas at the beginning of the follow-up period. Rate ratios for urban Aboriginal men were particularly elevated for digestive system diseases (RR = 3.00; 95% CI: 2.09-4.30), all external causes of death (RR = 2.80; 95% CI: 2.29-3.43)—notably motor vehicle accidents (RR = 3.51; 95% CI: 2.32-5.32) and, to a lesser extent, suicides (RR = 1.57; 95% CI: 1.04-2.38)—as well as for deaths due to infectious diseases (RR = 2.04; 95% CI: 1.33-3.11) including HIV/AIDS (RR = 2.03, CI: 1.22-3.39). With some exceptions (such as endocrine system diseases and suicide), rate ratios for Aboriginal men residing in metropolitan areas were similar to rate ratios for Aboriginal men residing in smaller urban centres.

Rate ratios for urban Aboriginal women were elevated for almost all major causes of death except breast cancer. For example, rate ratios were elevated for circulatory system diseases (RR = 1.93; 95% CI: 1.64-2.28) and all cancers combined (RR = 1.21; 95% CI: 1.03-1.42), the two most common causes of death. Rate ratios were particularly elevated for deaths due to infectious diseases (RR = 5.76; 95% CI: 3.68-9.01) including HIV/AIDS (RR = 10.65; 95% CI: 4.56-24.88), digestive system diseases (RR = 4.82; 95% CI: 3.67-6.34), external causes (RR = 3.37; 95% CI: 2.59-4.37)—notably motor vehicle accidents (RR = 4.13; 95% CI: 2.46-6.93)—and endocrine system diseases such as diabetes mellitus (RR = 2.61; 95% CI: 1.73-3.94). With some exceptions, rate ratios for Aboriginal women residing in metropolitan areas were higher than for Aboriginal women residing in smaller urban centres.

In Table 5, deaths are also categorized as smoking-related, alcohol-related, drug-related or amenable to medical care.^28,29^ Compared to urban non-Aboriginal men and women, rates for smoking-related causes (accounting for 15% and 7% of the total ASMR among urban Aboriginal men and women respectively) were elevated for urban Aboriginal men (RR = 1.46; 95% CI: 1.17-1.82) and women (RR = 1.36; 95% CI: 1.04-1.78). Rates for alcohol-related causes were considerably higher for urban Aboriginal men (RR = 4.55; 95% CI: 3.14-6.61) and women (RR = 11.44; 95% CI: 8.02-16.34), and rates for drug-related deaths were also significantly higher for Aboriginal men (RR = 3.71; 95% CI: 2.22-6.22) and women (RR = 6.43; 95% CI: 4.26-9.73). Rates of premature death (before the age of 75 years) due to causes considered amenable to medical intervention (for example, those due to breast and cervical cancer, infectious diseases, cerebrovascular disease, pneumonia or influenza) were also significantly higher for urban Aboriginal adults of both sexes.

Within the urban Aboriginal adult population, men were more likely than women to die from smoking-related causes (ASMR = 130 per 100 000 person-years at risk versus 58), but less likely to die from causes amenable to medical care (69 versus 92), a similar pattern to that of the non-Aboriginal population. The risks of dying from alcohol-related causes were slightly elevated for urban Aboriginal men compared to urban Aboriginal women (42 versus 34), a different pattern than for the non-Aboriginal population, where men had a much higher risk than did women (9 versus 3) (Table 4, Appendix Table D).

Table 6 shows unadjusted and adjusted all-cause mortality hazard ratios that compare urban Aboriginal adults to their non-Aboriginal counterparts. Urban Aboriginal men and women both had elevated hazard ratios (1.60 and 2.00, respectively); after controlling for community size, lone parenthood, educational attainment, income adequacy, occupation skill level, work status, and immigration, the hazard ratios were reduced to 1.22 and 1.68, respectively, suggesting that 63% (for men) and 32% (for women) of the differences in hazard ratios could be explained by those socio-economic variables.

## Discussion

This is the first in-depth study to examine mortality patterns for a large number of Aboriginal adults living in urban Canada. It is important to stress that place of residence and all demographic and socio-economic characteristics were measured only at baseline (June 4, 1991) and do not necessarily reflect the situation later in the follow-up period. Research shows that the Aboriginal population tends to move more frequently than the non-Aboriginal population.^6^ For example, about 70% of the Aboriginal population (all ages) residing in metropolitan areas changed residences between 1991 and 1996, with 45% moving within the same community.^6^

In this cohort, urban Aboriginal adults had higher mortality rates, shorter life expectancy and lower probability of survival to age 75 years compared to urban non-Aboriginal adults. This pattern of higher mortality is consistent with that previously observed for Registered Indians residing in Winnipeg,^19^ Vancouver^20^ and Canada as a whole.^14^

The higher mortality rates for Aboriginal people are thought to be the product of a wide range of social determinants, experienced from early childhood to old age, that influence health in complex and dynamic ways.^34,35^ Our study demonstrates that socio-economic variables were an important contributor to the elevated mortality rates of urban Aboriginal adults, especially for urban Aboriginal men.

Results by major causes of death revealed different patterns of risks. Compared to urban non-Aboriginal cohort members, rate ratios for urban Aboriginal adults were particularly elevated for some causes of death such as digestive system diseases, motor vehicle collisions, alcohol- and drug-related diseases and HIV/AIDS, while rate ratios for other causes, such as all cancers combined, were either similar or only slightly elevated. In such cases, rate ratios were generally similar between Aboriginal adults living in metropolitan areas and those living in smaller urban centres.

Circulatory system diseases were the most common cause of death among urban Aboriginal adults aged 25 years or older, accounting for 32% and 29% of all deaths for urban Aboriginal men and women, respectively. The majority of these deaths were due to ischemic heart disease. The relative risk of deaths due to circulatory system diseases were elevated for urban Aboriginal adults, as was found for Registered Indians in British Columbia.^20^ A study of Ontario First Nations found that the rate of hospital admission for ischemic heart disease rose dramatically from 1981 to 1997;^36^ some participants in that study may have moved to cities to obtain the use of specialized health care services that were not available in rural or remote settings.

All cancer deaths represented about one in four deaths among urban Aboriginal adults. Compared to urban non-Aboriginal adults, rate ratios for all-cancer mortality were not elevated for urban Aboriginal men and only slightly elevated for urban Aboriginal women, a similar finding to that for Registered Indians in British Columbia.^20^ However, grouping of all cancers together might mask important differences, as previous research has shown that Aboriginal people are at increased risk for certain cancers but not for others.^37-41^ Limited sample size prevented a detailed analysis of all types of cancer in this study, but our results show that the risk of cancer of the trachea, bronchus and lung was higher among urban Aboriginal adults, specifically those residing in metropolitan areas. Smoking prevalence, a risk factor for lung and other cancers, was more than twice as high among urban Aboriginal people aged 15 years or older compared to that of urban non-Aboriginal persons (43% versus 21%).^42^

Other studies have shown that the HIV/AIDS epidemic is particularly acute among Aboriginal people, especially among the young.^20,43^ Results from our study agree: rate ratios for HIV/AIDS mortality were more than twice for Aboriginal men and more than 10 times for Aboriginal women. Among Registered Indians in British Columbia, the rate of deaths due to HIV disease more than doubled from 1993 to 2006.^20^

The risk of dying from external injuries such as motor vehicle accidents and suicide was higher among urban Aboriginal adults than among urban non-Aboriginal adults. Other studies have also shown that Registered Indians and Aboriginal people in general are more likely to die from these causes than are other Canadians.^20, 44, 45^ A detailed breakdown of the different types of external causes of deaths was not possible due to the relatively small number of urban Aboriginal adults in the cohort, but the risk of dying from an external cause appeared greater for urban Aboriginal adults living in metropolitan areas than for those living in smaller urban centres. External causes of death accounted for a smaller proportion of all-cause mortality in this study compared to other studies, in part because our cohort followed people aged 25 years or older, whereas external injury deaths are most common among younger people.^44,45^ Because our study excluded the population under the age of 25 years, suicide rates reported in this study also failed to demonstrate the full extent of this problem as the mean age of deaths due to suicide was 27 years among Aboriginal people in Manitoba compared to 45 years for other Manitobans.^46^

The risk of dying from smoking-related diseases was higher among urban Aboriginal cohort members but the relative risk was not as high as for some other causes of death. In comparison, the relative risk of dying from alcohol-related diseases was considerably higher among urban Aboriginal adults (especially women) compared to urban non-Aboriginal adults. Other studies have shown that Registered Indians have a higher relative risk of dying of alcohol-related diseases compared to non-Aboriginal people.^17,20,47^ Despite this increased relative risk, deaths among urban Aboriginal adults due to alcohol-related diseases accounted for a smaller proportion of all deaths than did deaths due to smoking-related diseases.

Deaths prior to age 75 years that were amenable to medical care were elevated for urban Aboriginal adults compared to urban non-Aboriginal adults. Although the cause for this increased risk is not known, a 2004 study found that the proportion of persons who reported having a regular doctor did not vary between Aboriginal and non-Aboriginal persons living in urban Canada, but that urban Aboriginal people were more likely to report unmet health care needs than their non-Aboriginal counterparts.^42^

### Strengths and limitations

The large size of the Canadian census mortality follow-up study provides an opportunity to examine mortality patterns for urban Aboriginal adults. However, to be eligible for the study, and to be successfully linked, a person must have been enumerated by the 1991 long-form census and must have been a tax filer for the year 1990 or 1991. Thus any individual who did not file a tax return (under Section 87 of the *Indian Act*, Registered Indians are entitled to a tax exemption for income earned or considered to be earned on a reserve^48^) or who was in a long-term care facility, senior's residence or prison could not be included in the cohort. Despite this limitation, we found no major differences in demographic and socio-economic characteristics between eligible census respondents and those successfully linked to the name file.

Compared to life tables for all Canada (for 1995–1997), at age 25 years the entire cohort had remaining life expectancy 1 year longer for men, and 2 years longer for women.

Ascertainment of deaths was estimated to be slightly lower among Aboriginal persons (95% to 96%) compared to the cohort as a whole (97%). This would result in a slight downward bias in calculated mortality rates for the urban Aboriginal population, so the true extent of the disparities compared to the non-Aboriginal cohort could be slightly larger than indicated in this study.

Since a question on Aboriginal self-identity was not part of the 1991 census, this study defined the urban Aboriginal population on the basis of Aboriginal ancestry, Registered Indian status and/or membership in an Indian band or First Nation. This definition undoubtedly excluded many persons who would have self-identified as Aboriginal. According to the 1996 census results concerning self-identification with an Aboriginal group, about 8% of the self-identifying Aboriginal population did not report any Aboriginal ancestry,^32^ although some of the latter may have been Registered Indians or members of an Indian band or First Nation.

Studies have shown differences in health indicators for First Nations, Inuit and Métis.^10^ Since this study grouped First Nations, Inuit and Métis together, intra-group differences were obscured. Moreover, the results may not be reflective of Inuit living in urban areas since Inuit made up only 3% of the Aboriginal cohort.

## Conclusion

Until this study, only limited information on the mortality of the urban Aboriginal people of Canada was available. We found that mortality rates were higher for urban Aboriginal adults compared to urban non-Aboriginal adults. Circulatory system disease deaths and cancer deaths were the most common cause of death for urban Aboriginal and non-Aboriginal adults. However, relative risks were particularly elevated for some causes of death such as digestive system diseases, motor vehicle collisions, alcohol- and drug-related diseases and HIV/AIDS. In agreement with other research, our results also demonstrated that socio-economic status played an important role in explaining these disparities.

## Acknowledgments

Major funding for this study was provided by the Canadian Population Health Initiative, part of the Canadian Institute for Health Information. We would also like to acknowledge the key importance of Canada's provincial and territorial registrars of vital statistics, who provide the death data for the Canadian Mortality Data Base.

The views expressed in this article are those of the authors and do not necessarily reflect the views of the above-named organizations or of the institutions with which they are affiliated.

## Figures and Tables

**Table 1:** Characteristics of urban Aboriginal and urban non-Aboriginal cohort members by place of residence and sex, non-institutional population aged 25 years or older at baseline, Canada, 1991

	Aboriginal			Non-Aboriginal		

	All urban areas	Metropolitan areas^a^	Smaller urban centres^b^	All urban areas	Metropolitan areas^a^	Smaller urban centres^b^
**Both sexes**						
Number	16 300	10 400	5 900	2 062 700	1 633 600	429 100
Age 25-44 (%)	73	73	73	54	55	53
Age 65+ (%)	5	5	5	15	15	17
Married or common law (%)	62	60	67	73	72	76
Lone parent (%)	14	14	14	5	5	5
Less than high school diploma (%)	44	42	46	31	30	36
University degree (%)	5	6	3	16	17	10
Employed (%)	56	57	55	67	67	64
Two lowest income quintiles (%)	61	61	62	36	37	36
Activity limitation (%)	15	15	15	10	10	12
**Men**						
Number	6 900	4 400	2 500	1 013 300	799 800	213 400
Age 25-44 (%)	71	72	71	54	54	52
Age 65+ (%)	5	4	5	14	14	16
Married or common law (%)	67	64	73	79	78	82
Lone parent (%)	3	3	4	2	2	2
Less than high school diploma (%)	45	43	47	31	29	36
University degree (%)	5	7	2	18	19	12
Employed (%)	65	66	64	74	75	71
Two lowest income quintiles (%)	57	56	58	33	34	33
Activity limitation (%)	16	16	16	10	10	12
**Women**						
Number	9 400	6 000	3 400	1 049 400	833 700	215 700
Age 25-44 (%)	74	74	74	55	55	55
Age 65+ (%)	5	5	6	17	16	18
Married or common law (%)	59	56	63	68	67	70
Lone parent (%)	22	23	22	8	8	8
Less than high school diploma (%)	43	42	45	32	31	36
University degree (%)	5	6	3	13	15	9
Employed (%)	50	51	49	60	61	57
Two lowest income quintiles (%)	65	64	65	39	39	39
Activity limitation (%)	14	14	14	10	10	11

Source: 1991–2001 Canadian census mortality follow-up study.

a Population ≥ 100 000

b Population ≥ 10 000

**Table 2:** Remaining life expectancy at age 25 years and probability of survival to age 75 years (conditional on surviving to age 25 years) for urban Aboriginal adults and urban non-Aboriginal adults by place of residence and sex, non-institutional population aged 25 years or older at baseline, Canada, 1991–2001

	Total	95% CI	Men	95% CI	Women	95% CI
**Life expectancy at age 25 years (years)**						
**Aboriginal**						
All urban areas	50.4	(49.7-51.1)	48.1	(47.1-49.1)	52.7	(51.7-53.7)
Metropolitan areas^a^	50.3	(49.4-51.2)	48.2	(46.9-49.5)	52.4	(51.1-53.7)
Smaller urban centres^b^	50.9	(49.8-52.0)	48.2	(46.6-49.8)	53.6	(52.1-55.0)
**Non-Aboriginal**						
All urban areas	56.0	(56.0-56.1)	52.8	(52.8-52.9)	59.2	(59.2-59.3)
Metropolitan areas^a^	56.2	(56.1-56.2)	53.0	(52.9-53.1)	59.3	(59.3-59.4)
Smaller urban centres^b^	55.5	(55.4-55.6)	52.2	(52.1-52.4)	58.8	(58.7-59.0)
**Probability of survival to age 75 years (%)**						
**Aboriginal**						
All urban areas	57.5	(54.8-60.1)	52.2	(48.2-56.3)	62.7	(59.2-66.2)
Metropolitan areas^a^	55.4	(52.0-58.7)	52.5	(47.5-57.6)	58.2	(53.6-62.7)
Smaller urban centres^b^	61.3	(57.1-65.6)	51.8	(44.9-58.6)	70.9	(65.5-76.3)
**Non-Aboriginal**						
All urban areas	72.0	(71.8-72.2)	64.5	(64.3-64.8)	79.5	(79.3-79.7)
Metropolitan areas^a^	72.4	(72.2-72.5)	65.0	(64.7-65.3)	79.7	(79.5-79.9)
Smaller urban centres^b^	70.7	(70.3-71.0)	62.9	(62.4-63.4)	78.5	(78.0-78.9)

Source: 1991–2001 Canadian census mortality follow-up study.

Abbreviations: CI, confidence interval.

a Population ≥ 100 000

b Population ≥ 10 000

**Table 3:** Deaths and mortality rate ratios, by age group at baseline, sex and place of residence, for urban Aboriginal adults compared to urban non-Aboriginal adults, non-institutional population aged 25 years or older at baseline, Canada, 1991–2001

Sex and age group at baseline	All urban areas			Metropolitan areas^a^			Smaller urban centres^b^		

	Deaths	RR	95% CI	Deaths	RR	95% CI	Deaths	RR	95% CI
**Men**									
Total, 25 + years	563	1.56	(1.43-1.70)	354	1.59	(1.43-1.77)	209	1.45	(1.26-1.66)
25 to 34	67	2.17	(1.71-2.77)	42	2.14	(1.58-2.91)	25	2.16	(1.45-3.22)
35 to 44	78	1.77	(1.41-2.21)	55	1.96	(1.50-2.56)	23	1.41	(0.93-2.13)
45 to 54	123	1.94	(1.62-2.32)	81	2.04	(1.64-2.54)	42	1.68	(1.24-2.28)
55 to 64	122	1.43	(1.20-1.71)	78	1.49	(1.19-1.86)	44	1.29	(0.96-1.74)
65 to 74	103	1.31	(1.08-1.58)	63	1.23	(0.96-1.58)	40	1.41	(1.03-1.93)
75+	70	1.27	(1.01-1.61)	35	1.27	(0.91-1.77)	35	1.23	(0.89-1.72)
**Women**									
Total, 25 + years	563	1.94	(1.78-2.11)	377	2.10	(1.89-2.32)	186	1.68	(1.44-1.97)
25 to 34	72	3.19	(2.52-4.04)	52	3.71	(2.81-4.89)	20	2.25	(1.43-3.53)
35 to 44	100	2.55	(2.09-3.11)	70	2.87	(2.27-3.64)	30	1.96	(1.36-2.83)
45 to 54	112	2.39	(1.98-2.88)	80	2.72	(2.18-3.39)	32	1.78	(1.25-2.53)
55 to 64	93	1.71	(1.39-2.10)	65	1.87	(1.46-2.39)	28	1.38	(0.95-2.00)
65 to 74	115	1.61	(1.34-1.94)	76	1.80	(1.44-2.26)	39	1.31	(0.95-1.79)
75+	71	1.13	(0.90-1.43)	34	0.92	(0.66-1.29)	37	1.41	(1.02-1.95)

Source: 1991–2001 Canadian census mortality follow-up study.

Note: The rate ratio for all ages combined has been age standardized.

Reference population (person-years at risk) for age standardization was taken from the Aboriginal age distribution (5-year age groups).Abbreviations: CI, confidence interval; RR, rate ratio.

a Population ≥ 100 000

b Population ≥ 10 000

**Table 4:** Deaths and age-standardized mortality rates per 100 000 person-years at risk for urban Aboriginal adults by sex and place of residence, non-institutional population aged 25 years or older at baseline, Canada, 1991–2001

	All urban areas			Metropolitan areas^a^			Smaller urban centres^b^		

	Deaths	ASMR	95% CI	Deaths	ASMR	95% CI	Deaths	ASMR	95% CI
**Men**									
All causes	563	875.4	(804.5-952.4)	354	880.9	(791.0-981.1)	209	860.0	(749.9-986.3)
Infectious diseases	22	31.8	(20.9-48.5)	17	39.3	(24.2-63.8)	5	19.3	(8.0-46.3)
HIV/AIDS	15	21.3	(12.8-35.4)	—	—		—	—	
Other infectious diseases	7	10.5	(4.9-22.4)	—	—		—	—	
Cancer	132	203.8	(171.5-242.3)	79	197.9	(157.7-248.4)	53	215.6	(164.3-282.9)
Trachea/bronchus/lung cancers	51	79.8	(60.4-105.4)	32	83.5	(58.2-119.8)	19	76.7	(48.7-120.9)
Other cancers	81	124.1	(99.6-154.6)	47	114.4	(85.5-153.1)	34	138.9	(99.0-194.9)
Endocrine diseases	16	24.2	(14.7-39.7)	7	16.5	(7.8-34.9)	9	36.1	(18.7-69.9)
Circulatory system	178	285.1	(245.6-331.0)	107	279.5	(229.6-340.3)	71	299.3	(236.5-378.9)
Ischemic heart disease	116	185.0	(153.8-222.5)	71	189.1	(148.6-240.8)	45	193.0	(143.6-259.4)
Other circulatory diseases	62	100.1	(77.7-128.9)	36	90.4	(64.5-126.7)	26	106.3	(72.0-157.0)
Respiratory diseases	39	67.5	(49.1-92.7)	22	63.4	(41.0-98.0)	17	71.0	(43.8-115.1)
Digestive system diseases	37	60.2	(42.0-86.2)	28	73.5	(49.5-109.1)	9	33.8	(17.5-65.3)
External causes	97	138.3	(113.3-168.8)	64	142.4	(111.4-182.0)	33	132.9	(94.4-187.1)
Suicide	23	32.3	(21.4-48.6)	17	37.0	(23.0-59.6)	6	23.7	(10.6-52.8)
Motor vehicle	23	33.2	(22.0-50.0)	14	31.7	(18.8-53.6)	9	37.0	(19.2-71.4)
Other external causes	51	72.9	(55.4-95.9)	33	73.7	(52.4-103.7)	18	72.1	(45.4-114.6)
All other causes	42	64.5	(47.5-87.6)	30	68.5	(47.8-98.2)	12	51.9	(29.3-91.9)
Smoking-related diseases	81	130.3	(104.4-162.5)	51	137.6	(103.3-183.1)	30	122.4	(85.3-175.8)
Alcohol-related diseases	29	41.7	(28.8-60.3)	20	44.8	(28.7-70.0)	9	36.0	(18.7-69.5)
Drug-related diseases	15	20.6	(12.4-34.2)	—	—		—	—	
Amenable to medical intervention (< 75 years)	48	68.6	(51.7-91.1)	30	66.3	(46.4-94.9)	18	72.7	(45.7-115.7)
**Women**									
All causes	563	615.9	(566.2-670.0)	377	657.3	(593.0-728.7)	186	559.4	(478.9-653.5)
Infectious diseases	20	20.9	(13.5-32.4)	12	20.0	(11.4-35.3)	8	23.6	(11.7-47.2)
HIV/AIDS	6	6.1	(2.8-13.6)	—	—		—	—	
Other infectious diseases	14	14.8	(8.7-25.0)	—	—		—	—	
Cancer	153	162.9	(139.0-190.9)	98	166.9	(136.8-203.6)	55	158.5	(121.5-206.8)
Trachea/bronchus/lung cancers	36	38.6	(27.9-53.6)	26	45.9	(31.2-67.5)	10	27.3	(14.6-50.9)
Breast cancers	26	27.1	(18.4-39.8)	14	23.1	(13.7-39.1)	12	34.7	(19.7-61.2)
Other cancers	91	97.2	(79.1-119.4)	58	97.9	(75.6-126.7)	33	96.5	(68.4-136.1)
Endocrine	23	25.2	(16.7-37.9)	17	28.6	(17.7-46.0)	6	18.6	(8.4-41.6)
Circulatory system	154	178.4	(151.4-210.3)	108	197.3	(162.3-240.0)	46	158.2	(111.3-224.9)
Ischemic heart disease	72	83.5	(65.7-106.2)	50	89.0	(67.2-117.8)	22	78.9	(47.7-130.7)
Other circulatory diseases	82	94.9	(75.8-119.0)	58	108.3	(82.6-142.2)	24	79.3	(48.6-129.4)
Respiratory diseases	34	37.9	(26.8-53.5)	23	41.5	(27.3-62.9)	11	30.0	(16.6-54.3)
Digestive system diseases	53	55.9	(42.7-73.2)	38	62.6	(45.5-86.1)	15	43.5	(26.2-72.5)
External causes	58	59.9	(46.3-77.5)	43	70.5	(52.2-95.1)	15	42.6	(25.7-70.8)
Suicide	14	14.3	(8.5-24.1)	—	—		—	—	
Motor vehicle	15	15.6	(9.4-26.0)	11	18.1	(10.0-32.6)	4	11.4	(4.3-30.4)
Other external (excluding suicide)	29	30.0	(20.8-43.1)	—	—		—	—	
Other external (including suicide)	43	44.2	(32.8-59.7)	32	52.4	(37.0-74.1)	11	31.2	(17.3-56.5)
All other causes	68	74.9	(58.8-95.3)	38	70.0	(50.1-97.8)	30	84.3	(58.9-120.8)
Smoking-related diseases	54	57.8	(44.3-75.6)	36	62.5	(45.0-86.8)	18	49.1	(30.8-78.0)
Alcohol-related diseases	33	34.2	(24.3-48.1)	24	39.3	(26.4-58.7)	9	25.4	(13.2-48.9)
Drug-related diseases	24	24.5	(16.4-36.6)	17	27.5	(17.1-44.3)	7	19.6	(9.3-41.1)
Amenable to medical intervention (< 75 years)	86	92.1	(74.5-113.8)	60	101.5	(78.8-130.8)	26	77.0	(52.4-113.4)

Source: 1991–2001 Canadian census mortality follow-up study.

Abbreviations: —, suppressed due to disclosure rules or not applicable; AIDS, acquired immune deficiency syndrome; ASMR, age-standardized mortality rates; CI, confidence interval; HIV, human immunodeficiency virus.

Reference population (person-years at risk) for age standardization was taken from the Aboriginal age distribution (5-year age groups).

a Population ≥ 100 000

b Population ≥ 10 000

**Table 5:** Age-adjusted rate ratios by major causes of death and by sex, for urban Aboriginal adults compared to urban non-Aboriginal adults in same urban classification, non-institutional population aged 25 years or older at baseline, Canada, 1991–2001

	All urban areas		Metropolitan areas^a^		Smaller urban centres^b^	

	RR	95% CI	RR	95% CI	RR	95% CI
**Men**						
All causes	1.56	(1.43-1.70)	1.59	(1.43-1.77)	1.45	(1.26-1.66)
Infectious diseases	2.04	(1.33-3.11)	2.19	(1.34-3.56)	2.89	(1.19-7.03)
HIV/AIDS	2.03	(1.22-3.39)	—		—	
Other infectious diseases	2.04	(0.96-4.37)	—		—	
Cancer	1.09	(0.92-1.30)	1.07	(0.85-1.34)	1.11	(0.85-1.46)
Trachea/bronchus/lung cancers	1.42	(1.08-1.88)	1.53	(1.06-2.19)	1.26	(0.80-1.99)
Other cancers	0.95	(0.76-1.18)	0.88	(0.66-1.18)	1.04	(0.74-1.46)
Endocrine diseases	1.42	(0.86-2.33)	0.98	(0.46-2.08)	2.00	(1.03-3.89)
Circulatory system	1.50	(1.29-1.74)	1.51	(1.24-1.84)	1.45	(1.14-1.83)
Ischemic heart disease	1.52	(1.26-1.83)	1.59	(1.25-2.03)	1.46	(1.08-1.96)
Other circulatory diseases	1.47	(1.14-1.89)	1.36	(0.97-1.91)	1.43	(0.96-2.11)
Respiratory diseases	1.72	(1.25-2.37)	1.68	(1.08-2.59)	1.62	(1.00-2.64)
Digestive system diseases	3.00	(2.09-4.30)	3.67	(2.47-5.45)	1.68	(0.86-3.25)
External causes	2.80	(2.29-3.43)	3.04	(2.37-3.89)	2.26	(1.60-3.20)
Suicide	1.57	(1.04-2.38)	1.91	(1.18-3.08)	0.96	(0.43-2.16)
Motor vehicle	3.51	(2.32-5.32)	3.67	(2.16-6.23)	2.96	(1.51-5.78)
Other external causes	3.76	(2.84-4.96)	3.92	(2.78-5.54)	3.32	(2.07-5.33)
All other causes	1.47	(1.08-2.00)	1.57	(1.09-2.25)	1.18	(0.66-2.09)
Smoking-related diseases	1.46	(1.17-1.82)	1.59	(1.19-2.11)	1.24	(0.86-1.78)
Alcohol-related diseases	4.55	(3.14-6.61)	4.81	(3.06-7.55)	4.22	(2.16-8.24)
Drug-related diseases	3.71	(2.22-6.22)	—		—	
Amenable to medical intervention (< 75 years)	1.80	(1.35-2.39)	1.65	(1.15-2.37)	2.39	(1.49-3.82)
**Women**						
All causes	1.94	(1.78-2.11)	2.10	(1.89-2.32)	1.68	(1.44-1.97)
Infectious diseases	5.76	(3.68-9.01)	5.25	(2.95-9.32)	8.08	(3.91-16.69)
HIV/AIDS	10.65	(4.56-24.88)	—		—	
Other infectious diseases	4.84	(2.84-8.24)	—		—	
Cancer	1.21	(1.03-1.42)	1.25	(1.02-1.52)	1.16	(0.88-1.51)
Trachea/bronchus/lung cancers	1.33	(0.96-1.85)	1.61	(1.10-2.38)	0.86	(0.46-1.61)
Breast cancers	0.91	(0.62-1.34)	0.77	(0.46-1.31)	1.21	(0.68-2.14)
Other cancers	1.29	(1.05-1.58)	1.30	(1.00-1.69)	1.26	(0.89-1.78)
Endocrine	2.61	(1.73-3.94)	3.00	(1.86-4.85)	1.83	(0.82-4.12)
Circulatory system	1.93	(1.64-2.28)	2.19	(1.80-2.67)	1.56	(1.10-2.22)
Ischemic heart disease	1.73	(1.36-2.21)	1.89	(1.43-2.50)	1.52	(0.91-2.52)
Other circulatory diseases	2.15	(1.71-2.69)	2.53	(1.93-3.32)	1.61	(0.98-2.63)
Respiratory diseases	1.91	(1.35-2.71)	2.15	(1.41-3.26)	1.39	(0.77-2.52)
Digestive system diseases	4.82	(3.67-6.34)	5.41	(3.92-7.46)	3.73	(2.23-6.27)
External causes	3.37	(2.59-4.37)	4.12	(3.04-5.58)	2.09	(1.25-3.50)
Suicide	2.46	(1.45-4.19)	—		—	
Motor vehicle	4.13	(2.46-6.93)	5.24	(2.87-9.59)	2.22	(0.82-6.06)
Other external (excluding suicide)	3.65	(2.53-5.28)	—		—	
Other external (including suicide)	3.16	(2.33-4.28)	3.84	(2.70-5.46)	2.04	(1.12-3.73)
All other causes	2.63	(2.06-3.36)	2.43	(1.74-3.40)	3.10	(2.15-4.46)
Smoking-related diseases	1.36	(1.04-1.78)	1.50	(1.08-2.08)	1.07	(0.67-1.71)
Alcohol-related diseases	11.44	(8.02-16.34)	12.87	(8.49-19.50)	9.38	(4.70-18.73)
Drug-related diseases	6.43	(4.26-9.73)	7.40	(4.53-12.08)	4.65	(2.14-10.10)
Amenable to medical intervention (< 75 years)	1.99	(1.61-2.47)	2.20	(1.71-2.85)	1.65	(1.11-2.43)

Source: 1991–2001 Canadian census mortality follow-up study.

Abbreviations: —, suppressed due to disclosure rules or not applicable; AIDS, acquired immune deficiency syndrome; ASMR, age-standardized mortality rates; CI, confidence interval; HIV, human immunodeficiency virus.

Reference population (person-years at risk) for age standardization was taken from the Aboriginal age distribution (5-year age groups).

a Population ≥ 100 000

b Population ≥ 10 000

**Table 6:** Adjusted and unadjusted all-cause mortality hazard ratios for Aboriginal and non-Aboriginal adults residing in all urban areas, by sex, non-institutional population aged 25 years or older at baseline, Canada, 1991–2001

Characteristic at baseline	Men				Women			

	Unadjusted		Adjusted		Unadjusted		Adjusted	

	Hazard ratio	95% CI	Hazard ratio	95% CI	Hazard ratio	95% CI	Hazard ratio	95% CI
**Aboriginal**								
Yes	1.60	(1.47-1.73)	1.22	(1.13-1.33)	2.00	(1.84-2.17)	1.68	(1.55-1.83)
No (ref)	1.00	...	1.00	...	1.00	...	1.00	...
Age (years)	1.10	(1.10-1.10)	1.09	(1.09-1.09)	1.10	(1.10-1.10)	1.09	(1.09-1.09)
**Lone parent**								
Yes	…	...	1.04	(0.99-1.08)	…	...	1.10	(1.07-1.13)
No (ref)	…	...	1.00	...	…	...	1.00	...
**Place of residence**								
Metropolitan areas^a^	…	...	1.01	(1.00-1.03)	…	...	0.99	(0.98-1.01)
Smaller urban centres^b^	…	...	1.00	...	…	...	1.00	...
**Highest educational attainment**								
Less than high school diploma	…	...	1.35	(1.32-1.39)	…	...	1.24	(1.19-1.28)
High school diploma	…	...	1.22	(1.19-1.25)	…	...	1.15	(1.11-1.19)
Post-secondary diploma	…	...	1.09	(1.06-1.13)	…	...	1.07	(1.03-1.11)
University degree (ref)	…	...	1.00	...	…	...	1.00	...
**Income adequacy quintile**								
Quintile 1 - lowest	…	...	1.41	(1.38-1.44)	…	...	1.30	(1.27-1.33)
Quintile 2	…	...	1.18	(1.16-1.20)	…	...	1.13	(1.10-1.15)
Quintile 3	…	...	1.10	(1.07-1.12)	…	...	1.08	(1.05-1.11)
Quintile 4	…	...	1.04	(1.01-1.06)	…	...	1.04	(1.01-1.07)
Quintile 5 - highest (ref)	…	...	1.00	...	…	...	1.00	...
**Occupation - skill-based categories**								
Professional (ref)	…	...	1.00	...	…	...	1.00	...
Managerial	…	...	0.99	(0.95-1.03)	…	...	1.07	(0.99-1.15)
Skilled/Technical/Supervisory	…	...	1.09	(1.05-1.13)	…	...	1.10	(1.04-1.16)
Semi-skilled	…	...	1.19	(1.14-1.23)	…	...	1.11	(1.05-1.17)
Unskilled	…	...	1.27	(1.22-1.33)	…	...	1.18	(1.11-1.26)
No occupation	…	...	1.29	(1.24-1.34)	…	...	1.29	(1.22-1.36)
**Work status**								
Unemployed (ref)	…	...	1.00	...	…	...	1.00	...
Employed	…	...	0.82	(0.79-0.85)	…	...	0.89	(0.84-0.94)
Not in labour force	…	...	1.16	(1.11-1.20)	…	...	1.09	(1.03-1.16)
**Place of birth**								
Canada (ref)	…	...	1.00	...	…	...	1.00	...
Overseas	…	...	0.74	(0.73-0.75)	…	...	0.84	(0.83-0.86)

Source: 1991-2001 Canadian census mortality follow-up study.

Abbreviations: ..., not applicable; CI, confidence interval; ref, reference.

a Population ≥ 100 000

b Population ≥ 10 000

## Appendices

### Table A

**Table:** Long-form census respondents, cohort members, linkage rate to name file, deaths ascertained, and person-years at risk, non-institutional population aged 25 years or older at baseline, 1991

	Census respondents (n)	Study cohort (n)	Linkage rate to name file (%)	Deaths ascertained (n)	Person years
**Aboriginal population**					
**All urban areas**					
Total	25 500	16 300	64	1 126	166 570
Men	11 300	6 900	61	563	69 580
Women	14 200	9 400	66	563	96 990
**Metropolitan areas^a^**					
Total	15 800	10 400	66	731	106 030
Men	7 000	4 400	63	354	44 610
Women	8 800	6 000	68	377	61 410
**Smaller urban centres^b^**					
Total	9 700	5 900	61	395	60 540
Men	4 300	2 500	58	209	24 970
Women	5 400	3 400	64	186	35 570
**Non-Aboriginal**					
**All urban areas**					
Total	2 644 400	2 062 700	78	192 932	20 844 280
Men	1 270 400	1 013 300	80	111 126	10 145 220
Women	1 373 900	1 049 400	76	81 806	10 699 060
**Metropolitan areas^a^**					
Total	2 098 600	1 633 600	78	148 482	16 528 930
Men	1 007 700	799 800	79	84 836	8 022 930
Women	1 090 900	833 700	76	63 646	8 506 000
**Smaller urban centres^b^**					
Total	545 800	429 100	79	44 450	4 315 350
Men	262 700	213 400	81	26 290	2 122 290
Women	283 000	215 700	76	18 160	2 193 060

Source: 1991–2001 Canadian census mortality follow-up study.

Note: Census population counts rounded to nearest 100, person years rounded to the nearest 10.

a Population ≥ 100 000

b Population ≥ 10 000

### Table B

**Table:** Demographic and socio-economic characteristics at baseline of the in-scope (eligible) urban Aboriginal census respondents compared to urban Aboriginal cohort members, by sex and place of residence, non-institutional population aged 25 years or older at baseline, 1991

	All urban areas			Metropolitan areas^a^			Smaller urban centres^b^		

	In-scope %	Cohort %	Ratio	In-scope %	Cohort %	Ratio	In-scope %	Cohort %	Ratio
**Men**									
Number	49 100	6 900		33 100	4 400		16 000	2 500	
**Age group (years)**									
25 to 34	43	42	0.97	43	42	0.97	42	41	0.98
35 to 44	28	30	1.04	28	30	1.04	28	29	1.05
45 to 54	16	16	1.04	16	16	1.03	15	16	1.07
55 to 64	8	8	0.95	8	8	0.97	9	8	0.91
65 to 74	3	3	1.01	3	3	1.05	3	3	0.93
75 +	2	1	0.79	1	1	0.78	2	2	0.78
**Marital status**									
Single (never married)	26	23	0.88	28	25	0.90	21	18	0.84
Common-law	19	17	0.93	18	16	0.90	21	20	0.96
Married	42	50	1.17	41	48	1.17	45	53	1.17
Previously married	13	10	0.79	13	11	0.82	13	10	0.74
**Lone parent**									
Yes	96	97	1.01	96	97	1.01	96	96	1.01
No	4	3	0.83	4	3	0.79	4	4	0.87
**Educational attainment**									
Less than high school diploma	47	45	0.94	45	43	0.96	51	47	0.91
High school diploma	38	40	1.05	38	39	1.03	38	41	1.09
Post-secondary diploma	9	10	1.08	10	11	1.06	8	9	1.15
University degree	5	5	1.00	6	7	1.03	2	2	1.01
**Labour force status**									
Employed	61	65	1.06	62	66	1.05	59	64	1.09
Unemployed	17	16	0.94	15	14	0.92	19	18	0.95
Not in labour force	22	20	0.88	22	20	0.91	22	18	0.81
**Income quintile**									
Quintile 1 - lowest	39	34	0.88	39	34	0.89	38	33	0.86
Quintile 2	21	23	1.06	21	21	1.01	22	25	1.16
Quintile 3	17	19	1.12	17	19	1.12	16	18	1.11
Quintile 4	14	16	1.07	14	16	1.10	14	15	1.02
Quintile 5 - highest	9	9	1.03	9	9	1.07	9	9	0.97
**Activity limitation**									
Not stated	2	1	0.59	2	1	0.72	2	1	0.39
No	82	83	1.02	82	83	1.01	82	84	1.02
Yes	17	16	0.96	17	16	0.96	16	16	0.95
**Women**									
Number	65 500	9 400		43 700	6 000		21 800	3 400	
**Age group (years)**									
25 to 34	42	44	1.04	43	44	1.03	41	44	1.05
35 to 44	29	30	1.03	29	30	1.01	28	30	1.07
45 to 54	15	15	0.99	15	15	1.00	15	14	0.97
55 to 64	8	7	0.85	8	7	0.91	8	6	0.75
65 to 74	4	4	0.85	4	4	0.86	5	4	0.83
75 +	2	1	0.75	1	1	0.82	2	2	0.65
**Marital status**									
Single (never married)	20	19	0.95	21	20	0.95	18	17	0.95
Common-law	16	16	1.01	15	15	0.97	17	17	1.06
Married	40	43	1.08	38	41	1.08	42	46	1.08
Previously married	25	22	0.91	25	23	0.93	24	20	0.86
**Lone parent**									
Yes	23	22	0.95	23	23	0.96	23	22	0.93
No	77	78	1.01	77	77	1.01	77	78	1.02
**Educational attainment**									
Less than high school diploma	46	43	0.93	45	42	0.93	50	45	0.92
High school diploma	34	36	1.05	35	37	1.05	32	35	1.07
Post-secondary diploma	15	16	1.08	15	16	1.06	15	17	1.12
University degree	5	5	1.07	5	6	1.12	3	3	0.99
**Labour force status**									
Employed	46	50	1.10	47	51	1.08	43	49	1.14
Unemployed	11	11	0.98	10	10	0.97	13	13	0.98
Not in labour force	43	39	0.90	43	40	0.92	44	38	0.87
**Income quintile**									
Quintile 1 - lowest	47	43	0.93	47	44	0.93	46	42	0.92
Quintile 2	20	21	1.05	20	20	1.03	21	23	1.08
Quintile 3	15	16	1.06	14	15	1.06	15	16	1.05
Quintile 4	11	12	1.08	11	12	1.11	11	11	1.04
Quintile 5 - highest	8	8	1.08	8	8	1.09	7	8	1.08
**Activity limitation**									
Not stated	1	0	0.54	1	0	0.51	1	0	0.60
No	84	85	1.02	84	85	1.02	84	86	1.02
Yes	15	14	0.91	15	14	0.92	16	14	0.90

Source: 1991 census and 1991–2001 Canadian census mortality follow-up study.

a Population ≥ 100 000

b Population ≥ 10 000

### Table C

**Table:** Demographic and socio-economic characteristics of the in-scope (eligible) urban non-Aboriginal census respondents compared to urban non-Aboriginal cohort members, by sex and place of residence, non-institutional population aged 25 years or older at baseline, 1991

	All urban areas			Metropolitan areas^a^			Smaller urban centres^b^		

	In-scope %	Cohort %	Ratio	In-scope %	Cohort %	Ratio	In-scope %	Cohort %	Ratio
**Men**									
Number	6 470 600	1 013	300	5 159 500	799	800	1 311 100	213	400
**Age group (years)**									
25 to 34	29	28	0.95	30	28	0.94	27	26	0.97
35 to 44	26	26	1.02	26	26	1.01	26	26	1.03
45 to 54	18	18	1.03	18	18	1.03	17	18	1.02
55 to 64	14	14	1.03	13	14	1.04	14	14	1.01
65 to 74	9	10	1.04	9	10	1.05	11	11	1.01
75 +	5	5	0.97	4	4	0.97	6	5	0.93
**Marital status**									
Single (never married)	16	14	0.84	17	15	0.84	13	11	0.85
Common-law	7	7	0.90	7	6	0.90	8	7	0.88
Married	67	72	1.08	66	72	1.08	70	75	1.07
Previously married	9	7	0.81	9	7	0.80	9	8	0.81
**Lone parent**									
Yes	98	98	1.00	98	98	1.00	98	98	1.00
No	2	2	0.86	2	2	0.86	2	2	0.85
**Educational attainment**									
Less than high school diploma	32	31	0.97	30	29	0.97	38	36	0.96
High school diploma	38	38	1.01	37	37	1.00	40	40	1.02
Post-secondary diploma	13	14	1.03	14	14	1.02	12	12	1.04
University degree	17	18	1.03	19	19	1.03	11	12	1.05
**Labour force status**									
Employed	72	74	1.02	73	75	1.02	69	71	1.03
Unemployed	6	6	0.91	6	6	0.90	7	6	0.93
Not in labour force	21	20	0.96	20	20	0.96	24	23	0.95
**Income quintile**									
Quintile 1 - lowest	16	14	0.87	17	14	0.87	15	13	0.88
Quintile 2	20	19	0.99	20	19	0.99	19	19	0.99
Quintile 3	21	21	1.02	21	21	1.02	21	22	1.03
Quintile 4	21	22	1.04	21	22	1.04	22	23	1.04
Quintile 5 - highest	22	23	1.05	22	23	1.05	22	23	1.03
**Activity limitation**									
Not stated	1	0	0.77	1	0	0.74	1	1	0.88
No	89	89	1.01	89	90	1.01	86	87	1.01
Yes	11	10	0.96	10	10	0.97	13	12	0.94
**Women**									
Number	6 983 500	1 049 400	1.00	5 574 600	833 700	1.00	1 408 900	215 700	1.00
**Age group (years)**									
25 to 34	27	29	1.04	28	29	1.03	26	28	1.09
35 to 44	25	26	1.08	25	26	1.07	24	26	1.10
45 to 54	16	17	1.01	16	17	1.01	16	16	1.00
55 to 64	13	12	0.89	13	12	0.90	14	12	0.85
65 to 74	11	10	0.91	11	10	0.92	12	11	0.86
75 +	7	6	0.89	7	6	0.90	8	7	0.89
**Marital status**									
Single (never married)	12	13	1.01	13	13	1.00	9	10	1.07
Common-law	6	6	1.00	6	6	1.00	6	6	1.01
Married	61	62	1.02	60	61	1.02	63	63	1.00
Previously married	21	20	0.95	21	20	0.94	21	21	0.97
**Lone parent**									
Yes	91	92	1.00	91	92	1.00	92	92	1.00
No	9	8	0.96	9	8	0.95	8	8	0.99
**Educational attainment**									
Less than high school diploma	35	32	0.91	34	31	0.91	40	36	0.89
High school diploma	34	36	1.03	34	35	1.03	34	36	1.04
Post-secondary diploma	18	19	1.08	18	19	1.07	18	19	1.10
University degree	12	13	1.08	14	15	1.07	8	9	1.14
**Labour force status**									
Employed	55	60	1.09	56	61	1.08	51	57	1.12
Unemployed	5	5	1.01	5	5	1.01	5	6	1.03
Not in labour force	40	35	0.88	39	34	0.88	44	38	0.86
**Income quintile**									
Quintile 1 - lowest	21	20	0.92	21	19	0.91	22	20	0.94
Quintile 2	21	20	0.96	21	20	0.97	20	19	0.94
Quintile 3	20	20	1.02	20	20	1.02	20	20	1.02
Quintile 4	19	20	1.05	19	20	1.05	19	20	1.04
Quintile 5 - highest	19	20	1.07	19	20	1.06	19	20	1.07
**Activity limitation**									
Not stated	1	0	0.89	1	0	0.87	0	0	0.97
No	88	89	1.01	89	90	1.01	87	88	1.02
Yes	11	10	0.90	11	10	0.91	13	11	0.89

Source: 1991 census and 1991–2001 Canadian census mortality follow-up study.

a Population ≥ 100 000

b Population ≥ 10 000

### Table D

**Table:** Deaths and age-standardized mortality rates per 100 000 person-years at risk for urban non-Aboriginal adults by sex and place of residence, non-institutional population aged 25 years or older at baseline, Canada, 1991–2001

	All urban areas			Metropolitan areas^a^			Smaller urban centres^b^		

	Deaths	ASMR	95% CI	Deaths	ASMR	95% CI	Deaths	ASMR	95% CI
**Men**									
All causes	111 126	561.7	(558.1-565.4)	84 836	553.2	(549.1-557.3)	26 290	592.8	(584.6-601.0)
Infectious diseases	1 875	15.6	(14.9-16.4)	1 654	18.0	(17.0-19.0)	221	6.7	(5.7-7.8)
HIV/AIDS	919	10.5	(9.8-11.2)	876	12.6	(11.7-13.5)	43	2.4	(1.8-3.3)
Other infectious diseases	956	5.1	(4.8-5.5)	778	5.4	(5.0-5.8)	178	4.2	(3.6-5.0)
Cancer	37 073	186.7	(184.7-188.8)	28 544	184.8	(182.5-187.1)	8 529	194.1	(189.5-198.7)
Trachea/bronchus/lung cancers	11 315	56.0	(54.9-57.1)	8 624	54.7	(53.5-55.9)	2 691	60.7	(58.3-63.3)
Other cancers	25 758	130.7	(129.0-132.5)	19 920	130.1	(128.1-132.1)	5 838	133.3	(129.6-137.2)
Endocrine diseases	3 463	17.1	(16.4-17.7)	2 647	16.8	(16.1-17.5)	816	18.0	(16.7-19.5)
Circulatory system	40 955	190.0	(188.0-192.0)	30 951	185.2	(183.0-187.4)	10 004	207.1	(202.6-211.6)
Ischemic heart disease	25 856	121.7	(120.1-123.3)	19 645	118.7	(117.0-120.5)	6 211	132.5	(128.9-136.2)
Other circulatory diseases	15 099	68.3	(67.1-69.5)	11 306	66.5	(65.2-67.8)	3 793	74.6	(72.0-77.2)
Respiratory diseases	9 390	39.2	(38.3-40.0)	6 971	37.8	(36.9-38.7)	2 419	43.8	(42.0-45.7)
Digestive system diseases	3 886	20.1	(19.4-20.8)	3 004	20.0	(19.3-20.8)	882	20.2	(18.7-21.7)
External causes	5 710	49.3	(47.9-50.8)	4 279	46.9	(45.3-48.4)	1 431	58.8	(55.5-62.4)
Suicide	2 063	20.5	(19.6-21.5)	1 547	19.4	(18.4-20.5)	516	24.6	(22.4-27.0)
Motor vehicle	1 031	9.4	(8.8-10.1)	746	8.7	(8.0-9.4)	285	12.5	(11.0-14.3)
Other external causes	2 616	19.4	(18.6-20.3)	1 986	18.8	(17.9-19.8)	630	21.7	(19.8-23.8)
All other causes	8 774	43.8	(42.8-44.8)	6 786	43.7	(42.6-44.9)	1 988	44.1	(41.9-46.5)
Smoking-related diseases	18 829	89.4	(88.0-90.8)	14 182	86.8	(85.3-88.3)	4 647	98.9	(95.8-102.0)
Alcohol-related diseases	1 433	9.2	(8.6-9.7)	1 145	9.3	(8.7-9.9)	288	8.5	(7.5-9.7)
Drug-related diseases	513	5.1	(5.6-5.1)	410	5.6	(5.1-6.2)	103	5.4	(4.4-6.6)
Amenable to medical intervention (< 75 years)	5 540	38.1	(37.0-39.2)	4 473	40.1	(38.8-41.4)	1 067	30.4	(28.5-32.5)
**Women**									
All causes	81 806	317.6	(314.9-320.2)	63 646	313.6	(310.7-316.6)	18 160	332.5	(326.6-338.6)
Infectious diseases	834	3.6	(3.3-4.0)	685	3.8	(3.5-4.2)	149	2.9	(2.4-3.6)
HIV/AIDS	54	0.6	(0.4-0.8)	51	0.7	(0.5-0.9)	3	0.2	(0.1-0.6)
Other infectious diseases	780	3.1	(2.8-3.3)	634	3.1	(2.9-3.5)	146	2.7	(2.2-3.3)
Cancer	27 256	134.3	(132.5-136.2)	21 495	133.6	(131.5-135.7)	5 761	137.2	(133.1-141.5)
Trachea/bronchus/lung cancers	5 687	29.1	(28.2-29.9)	4 433	28.4	(27.5-29.4)	1 254	31.7	(29.8-33.8)
Breast cancers	5 158	29.7	(28.8-30.6)	4 111	29.9	(28.9-31.0)	1 047	28.8	(26.8-30.9)
Other cancers	16 411	75.5	(74.2-76.9)	12 951	75.2	(73.7-76.8)	3 460	76.7	(73.7-79.9)
Endocrine	2 696	9.6	(9.2-10.1)	2 110	9.5	(9.0-10.0)	586	10.2	(9.2-11.2)
Circulatory system	30 369	92.4	(91.1-93.6)	23 298	90.0	(88.6-91.3)	7 071	101.4	(98.6-104.3)
Ischemic heart disease	16 007	48.2	(47.3-49.0)	12 328	47.1	(46.2-48.1)	3 679	52.0	(50.1-54.0)
Other circulatory diseases	14 362	44.2	(43.3-45.1)	10 970	42.8	(41.9-43.8)	3 392	49.3	(47.3-51.4)
Respiratory diseases	6 421	19.8	(19.2-20.4)	4 953	19.3	(18.7-20.0)	1 468	21.6	(20.3-22.9)
Digestive system diseases	3 070	11.6	(11.1-12.1)	2 376	11.6	(11.0-12.1)	694	11.7	(10.6-12.8)
External causes	2 995	17.8	(17.0-18.6)	2 273	17.1	(16.3-18.0)	722	20.4	(18.6-22.4)
Suicide	610	5.8	(5.3-6.3)	490	5.8	(5.3-6.4)	120	5.8	(4.8-7.0)
Motor vehicle	491	3.8	(3.4-4.2)	356	3.4	(3.1-3.9)	135	5.1	(4.2-6.2)
Other external (excluding suicide)	1 894	8.2	(7.7-8.7)	1 427	7.9	(7.3-8.4)	467	9.5	(8.4-10.7)
Other external (including suicide)	2 504	14.0	(13.3-14.7)	1 917	13.6	(12.9-14.4)	587	15.3	(13.8-17.0)
All other causes	8 165	28.5	(27.7-29.2)	6 456	28.8	(27.9-29.7)	1 709	27.2	(25.6-28.9)
Smoking-related diseases	9 530	42.6	(41.6-43.6)	7 430	41.8	(40.7-42.9)	2 100	45.8	(43.6-48.2)
Alcohol-related diseases	484	3.0	(2.7-3.3)	382	3.1	(2.7-3.4)	102	2.7	(2.2-3.4)
Drug-related diseases	413	3.8	(3.4-4.2)	326	3.7	(3.3-4.2)	87	4.2	(3.4-5.3)
Amenable to medical intervention (< 75 years)	6 595	46.2	(45.0-47.4)	5 230	46.0	(44.7-47.4)	1 365	46.8	(44.2-49.6)

Source: 1991–2001 Canadian census mortality follow-up study.

Abbreviations: —, not applicable; AIDS, acquired immune deficiency syndrome; ASMR, age-standardized mortality rates; CI, confidence interval; HIV, human immunodeficiency virus.

Reference population (person-years at risk) for age standardization was taken from the Aboriginal age distribution (5-year age groups).

a Population ≥ 100 000

b Population ≥ 10 000
